# Preparatory Course in the Atlanta Medical College

**Published:** 1859-07

**Authors:** 


### EDITORIAL AND MISCELLANEOUS.

**PREPARATORY COURSE IN THE ATLANTA MEDICAL COLLEGE.**

The Faculty of this Institution, in view of the great favor with which the Prepara'ory Course of Instruction last winter has been received, and in view of the great advantages to be derived by the student from an elementary course embracing *an additional period of four months in each year (making eight months of instruction annually in the Atlanta Medical College,)* have determined to make it a permanent feature of the Institusion, and will, accordingly, resume their labors in that capacity the first of November, and continue until the first of March, and thus again we shall refer to the fact, that while some other Institutions are engaged in a senseless and contemptible clamor for the *regulation* of Medical education by power and influcnces, other than those belonging to an honorable and enlightened competition, the Atlanta Medical College, in a spirit of generous rivalry, and with the desire to use every reasonable effort to extend the opportunities of the Student for the acquisition of a higher grade of qualification, and with the earnest purpose of elevating the standard of Medical education, has gone on "proving her faith by her works," until her term of instruction has been *doubled* without any increase in the expense to the student—the additional course counting nothing towards graduation, but furnishing an opportunity to those who choose to avail themselves of it, of a greatly increased and gratuitous facility for acquiring medical knowledge. This course we are pleased to know has met the approbation of the medical men of our own section, (to whom alone we look for patronage,) as has been most conclusively shown by their sending up to the Institution, constantly increasing classes of those, whom, we shall not hesitate to say, are among the best young men in the South. In conclusion, we would remark, that if one-half of the labor consumed upon the part of one or two of the immediate competitors of this Institution, in attempts to disparage it, and in concealed and intriguing *correspondence* for its injury, was expended in increasing their own facilities, the results would be much more creditable to themselves, and redound, in a far higher degree, to their own prosperity.

The Class of the present year will *exceed* that of any previous year, by about one-fourth.

